# Comparison of blood carbonic anhydrase activity of athletes performing interval and continuous running exercise at high altitude

**DOI:** 10.1080/14756366.2018.1545768

**Published:** 2018-12-06

**Authors:** Murat Tas, Esra Senturk, Deniz Ekinci, Ramazan Demirdag, Veysal Comakli, Metin Bayram, Murat Akyuz, Murat Senturk, Claudiu T. Supuran

**Affiliations:** a Faculty of Sport Sciences, Manisa Celal Bayar University, Manisa, Turkey;; b School of Health Services, Agri Ibrahim Cecen University, Agri, Turkey;; c Faculty of Agriculture, Ondokuz Mayıs University, Samsun, Turkey;; d Physical Education Sports High School, Agri Ibrahim Cecen University, Agri, Turkey;; e Faculty of Pharmacy, Agri Ibrahim Cecen University, Agri, Turkey;; f Section of Pharmaceutical Chemistry, Neurofarba Department, University of Florence, Firenze, Italy

**Keywords:** Carbonic anhydrase, V02max, exercise training, enzyme inhibition

## Abstract

The effects of high-intensity interval and continuous exercise on erythrocytes carbonic anhydrase (CA, EC 4.2.1.1) activity levels were scarcely investigated up until now. Here we present a study focused on the CA activity from erythrocytes of athletes experiencing interval and continuous training for 6 weeks, during cold weather and at high altitude (> 1600 m). We observed a 50% increase in the blood CA activity at the second week after initiation of the training in both interval and continuos running groups, whereas the control group did not experience any variation in the enzyme activity levels. In the trained individuals a mild decrease in their body mass, BMI and an increased $V_{O_{2}\text{max}}$ were also observed. The CA activity returned at the basal values after 4–6 weeks after the training started, probably proving that a metabolic compensation occurred without the need of an enhanced enzyme activity. The unexpected 50% rise of activity for an enzyme which acts as a very efficient catalyst for CO_2_ hydration/bicarbonate dehydration, such as the blood CA, deserves further investigations for better understanding the physiologic basis of this phenomenon.

## 1. Introduction

Carbonic anhydrase (CA; carbonate hydrolyase, EC 4.2.1.1) enzyme, which exists commonly in living organisms, has various izoenzymes according to conditions and necessities of the medium. A new type of isoenzyme has been discovered almost for each year and now it has sixteen isoenzymes. It is one of the most studied enzymes and CA-I and CA-II are the most common isoenzymes^1^. A number of different CA isozymes have been described in higher vertebrates. Sixteen izozymes have been defined up to now, that differ in their subcellular place, catalytic activity and emotional different types of inhibitors. Some of these enzymes are cytolosolic (CA I, CA II, CA III, CA VII and CA XIII), others are membrane bound (CA IV, CA IX, CA XII and CA XIV), two are mitochondrial (CA VA and CA VB), and one is secreted in saliva (CA VI). It has been clarified that there is no expression of CA XV in human and other primates, but it is plentiful in rodents and other higher vertebrates^2^ ^,^ ^3^.

The enzyme has esterase activity besides its hydratase activity, but physiologically hydratase activity is of great importance. For instance, it plays an important role in the regulation of acid-base balance in the organisms. Where this balance is impaired, for example, in intraocular tension, therapeutic intervention to carbonic anhydrase enzyme activity is a common applied method. In this respect, compounds of carbonic anhydrase inhibitors gain a clinical importance^4^.

Interval training is a type of discontinuous physical training that involves a series of low- to high-intensity exercise workouts interspersed with rest or relief periods. The high-intensity periods are typically at or close to anaerobic exercise, while the recovery periods involve the activity of lower intensity. Interval training can be described as short periods of work followed by rest. The main aim is to improve speed and cardiovascular fitness. Interval training can refer to organisation of any cardiovascular workout (e.g. cycling, running, rowing, etc.), and is prominent in training routines for many sports. It is a technique particularly employed by runners, but athletes in many disciplines use this type of training^5^.

Continuous training is a type of physical training that involves activity without rest intervals. It is divided into three parts (1) slow continuous running, (2) fast continuous training and (3) variable pace described as follows:

1. Intensity will be low and heart beat 60–80% of HR_max_.
2. Intensity will be high and heart beat 85–95% of HR_max_, duration will be 15–20min.
3. It is the combination of both the runnings^6^. This type of training may be of high intensity, or moderate intensity with an extended duration, or fartlek training^7^.

Exercise modes noted as suitable for continuous training include indoor and outdoor cycling, jogging, running, walking, rowing, etc^8^.

$V_{O_{2}\text{max}}$ (maximal oxygen uptake) is an intense running pace which can be maintained for only about six minutes. This is the minimum speed for which the organism's $V_{O_{2}\text{max}}$ is reached (after a few minutes of exercise at this intensity); at higher paces, additional power is entirely delivered by anaerobic processes. At this pace, blood lactate in the muscles reaches levels around 8–10 mM.

The $V_{O_{2}\text{max}}$ of world class middle and long-distance runners may exceed 24 km/h (14.9 mph or about 4:00/mile pace), making this speed slightly comparable to 3000 m race pace. For many athletes, $V_{O_{2}\text{max}}$ may be slightly slower than 1500 m or mile race pace.

All these studies applied the so-called “moderate exercise intensity” as, at least, one of the exercise intensities in their physical activity programs. These exercise intensities were usually determined by a certain percentage of the individuals’ $V_{O_{2}\text{max}}$ or maximal heart rate (HR), but little evidence is available on why the exercise intensity was chosen and what energy substrates were used during exercise training at these intensities^9^. Some previous studies have shown that the FAT_max_ intensity usually occurs between 39% and 65% of $V_{O_{2}\text{max}}$ and varies according to the gender, body composition, training status, $V_{O_{2}\text{max}}$, and diet of the study participants^9–11^.

The aim of this study was measured human erythrocyte CA activity effects of some different exercise applications on college students.

## 2. Materials and methods

### 2.1. Participants

Twenty male university students, aged 20–22 years, were enrolled in this study. The inclusion criteria were males who had body mass index (BMI) > 21 kg/m^2^. Students who were afflicted by heart diseases, hypertension, pulmonary diseases, and diabetes; who needed orthopedic treatments; and who had neurological limitations to physical exercise were excluded. The exact details of the study were described to the participants prior to the baseline test, while a written informed consent to the study was obtained from each of them. This study was approved by the Ethics Committee of Ataturk University, Turkey.

### 2.2. Study design

Following the baseline test, the participants were randomly allocated into three groups: the interval exercise training group, continuous training group, and the control group. Those in these groups underwent 6 weeks of supervised exercise training. The participants in the control group were required to maintain their individual habits of physical activities and to refrain from engaging in any other forms of prescribed exercise training during the period of experimentation. Each participant’s body mass, height, and $V_{O_{2}\text{max}}$ were measured at the baseline, as well as after 6 weeks of the experimental period. The post-training tests and the last training session were separated by at least 1 day. Under the complete supervision of the researchers, all tests and training sessions were conducted in the exercise physiology laboratory and the sports grounds of Agri Ibrahim Cecen University Central Laboratory. Meanwhile, all the participants were required to maintain their normal diet during the period of experimentation.

### 2.3. Maximal oxygen uptake

Each participant’s $V_{O_{2}\text{max}}$ was measured by a graded treadmill walking/running test (Pulsar Cosmos Treadmill, Germany). After a warm-up period, the initial workload was set at the speed of 3.3 km/h at a 0% incline for 3 min. The second workload was executed at the speed of 6.3 km/h at a 0% incline for 1 min; the speed was then increased by 0.8 km/h per min. When the speed was increased up to 10 km/h, the incline was simultaneously increased by 1% per min as the speed still continued to increase by 0.8 km/h per min. This procedure continued until the participant had reached exhaustion, at which time the test was terminated. The criteria for measuring/were the following: a leveling off of $V_{O_{2}}$ despite increased workload, a respiratory exchange ratio (RER) equal to or higher than 1.05, and an exercise HR higher than 180 beats per min. $V_{O_{2}}$ and carbon dioxide production ($V_{{\text{CO}}_{2}}$) were measured by an open-circuit indirect gas analyser (Cortex Metalyzer II gas analyzer, Germany), which was calibrated with the standard gas prior to each test.

### 2.4. Carbonic anhydrase activity assay

Fresh human blood collected in tubes with EDTA was centrifuged at 2500×*g* for 15 min and the plasma and leukocyte coat were removed. The packed red cells were washed with KCl solution (0.16 M) three times and pelleted each time by centrifugation at 2500×*g*. The supernatant was discarded. The pelleted erythrocytes were hemolysed with 5 vol. of ice-cold water and centrifuged at 4 °C at 10,000×*g* for 30 min to remove the ghosts and any remaining intact cells^12–14^. Carbonic anhydrase activity was assayed by following the change in absorbance at 348 nm of 4-nitrophenylacetate (NPA) to 4-nitrophenylate ion over a period of 3 min at 25 °C using a spectrophotometer (ShimadzuUV-1800) according to the method described by Verpoorte et al.^15^ The enzymatic reaction, in a total volume of 3.0 ml, contained 1.4 ml 0.05 M Tris-SO_4_ buffer (pH 7.4), 1 ml 3 mM of 4-nitrophenylacetate, 0.5 ml H_2_O and 0.1 ml enzyme solution. A reference measurement was obtained by preparing the same cuvette without enzyme solution^16^ ^,^ ^17^.

### 2.5. Protein determination

Protein during the purification steps was determined spectrophotometrically at 595 nm according to the Bradford method, using bovine serum albumin as the standard^18^.

### 2.6. Statistical analyses

All the values were presented as mean ± SD. The paired Student *t* test was applied to test the changes in the measured variables within the groups. To evaluate the effects of exercise training, unpaired Student *t* test was used to compare the baseline data, as well as the changes in the measured variables after the interventions between the groups. A *p* value of <.05 was regarded as statistically significant. All the analyses were performed using the SPSS Version 11.5 for Windows (SPSS).

## 3. Results and discussion

CA, the enzyme that catalyses the reversible reaction involving the hydration–dehydration of CO_2_ and HCO_3_^−^, facilitates the transport of CO_2_ from the tissues to the lungs. Maximal aerobic capacity, determined from peak O_2_ uptake ($V_{O_{2}\text{peak}}$) was reduced. Kowalchuk et al.^19^ reported a lower $V_{O_{2}\text{peak}}$ but similar power output after acute infusion of acetazolamide compared with the uninhibited condition during 30 s of maximal-intensity exercise. This effect was not associated with any difference in plasma acid–base status before or immediately after the exercise bout, suggesting that CA inhibition may directly affect the exercise response^19^.

During exercise of progressively increasing intensity, a work rate is reached where, relative to O_2_ uptake ($V_{O_{2}}$), there is a disproportionate increase in CO_2_ output ($V_{{\text{CO}}_{2}}$) and ventilation [V_E_; i.e. ventilatory threshold (V_E_T)], as well as an increase in muscle and blood lactate (La^−^) concentration [La2]; i.e. lactate threshold (LaT). The V _E_T and LaT are typically observed at the same exercise intensity; however, a ‘‘cause-effect’’ relationship remains controversial^20^. Lactic acid (HLa) is almost completely dissociated at physiological pH (pK_a_ = 3.8). The H^+^ generated coincident with lactate anion formation is buffered primarily by HCO_3_^−^, resulting in the formation of CO_2_ and H_2_O at the tissues according to the net reaction:

$$H^{+}+{\text{HCO}}_{3}{}^{-}⇄{\text{CO}}_{2}+H_{2}O$$

The presence of CA speeds the dehydration of HCO_3_^−^, thus ensuring rapid equilibrium between CO_2_ species. Consequently, this increase in muscle CO_2_ production results in an increase in venous blood $P_{{\text{CO}}_{2}}$ and H^+^ concentration ([H^+^]), which contributes to the disproportionate increases in V_E_ and $V_{{\text{CO}}_{2}}$ relative to $V_{O_{2}}$ that are observed coincident with the V_ET_ ^21^. Acute CA inhibition with acetazolamide is associated with a lower $V_{{\text{CO}}_{2}}$ and a reduction in plasma [La^−^] ([La^−^]_pl_) during short-term maximal exercise^19^, both of which may be expected to affect theV_ET_ and/or the LaT, but the effect of acute acetazolamide administration on the exercise response to ramp exercise has not been examined^22^.

Prophylactic use of acetazolamide (AZ) has become a popular alternative to staged acclimatisation because it can be a less time-consuming method of reducing symptoms of acute mountain sickness (AMS). A very recent review revealed that AZ treated cohorts experience a reduction in time to exhaustion during both submaximal and maximal exercise performance at sea level. At altitude, AZ-treated cohorts showed widely variable submaximal and maximal exercise performance^23^.

**Figure 1.**
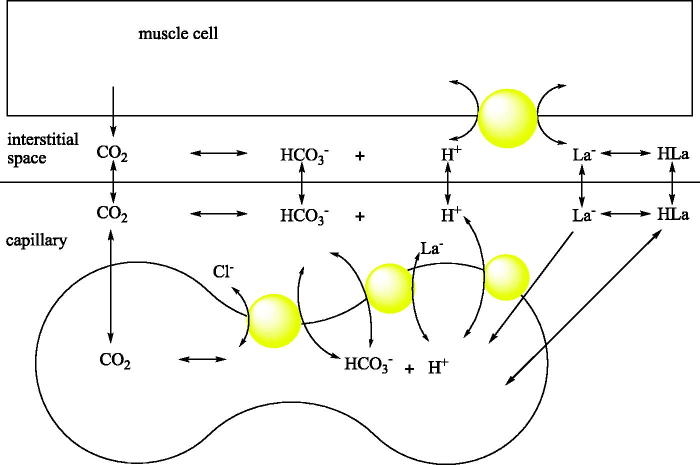
pH balance of the skeletal muscle and the involvement of blood CAs in the process.

The model showing the pH balance of the skeletal muscle and the role of CA is shown in Figure 1 ^23^. When an athlete ascends from sea level to moderate altitude, the shortage of oxygen (hypoxia) initially impairs endurance training and performance. After a few weeks at altitude, training and performance recover to some extent as the athlete adapts. If the athlete then returns to sea level, do the adaptations lead to enhancement of endurance performance? Coaches have long thought so, but studies aimed at this question appeared to be inconclusive, leading researchers to suspect that any benefit from adaptation to hypoxia was offset by the loss of endurance fitness consequent to the reduction in training intensity^24^.

**Figure 2.**
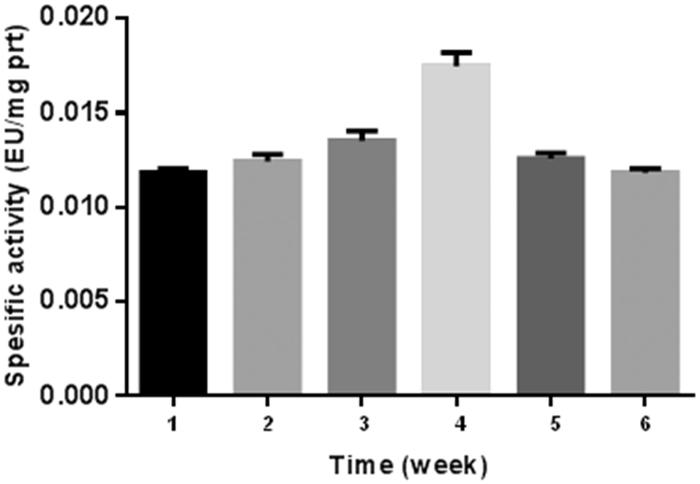
Interval training graph. **1:** control, **2:** 1st week before training, **3:** 1st week after training, **4:** 2nd week after training, **5:** 4th week after training, **6:** 6th week after training.

**Figure 3.**
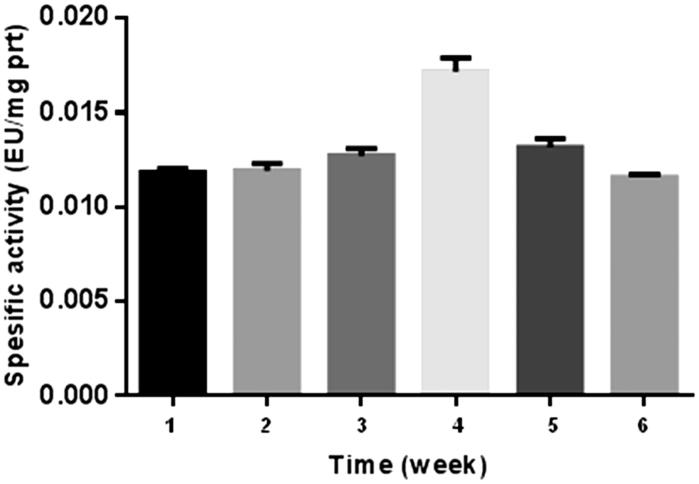
Continuous training graph. **1:** control, **2:** 1st week before training, **3:** 1st week after training, **4:** 2nd week after training, **5:** 4th week after training, **6:** 6th week after training.

The results of the present study are shown in Figures 2 and 3, and Tables 1 and 2. The results are based on the observations of seven participants in the interval exercise training group and seven participants in the continuous training group, whereas six participants were in the control group. As for those who successfully completed the training program, there were no reported physical injuries that were caused by physical activities during the training sessions. Because of this physiological importance, studies on this enzyme activity have recently been much popularised among the scientists^25^ ^,^ ^26^. The number of studies on CA enzyme activity has increased enormously^25–27^. In addition, some studies on the relationship between CA activity and exercise exist in literature^22^. Another study revealed that intensity of maximal fat oxidation in overweight young women, which had running exercise, compared to control groups^28^. In our study, it is clear from the results that exercise-induced effect in CA activity was recovered by 1640 m sea level and average temperature −15 °C. Figures 2 and 3 also show a comparison of CA activity among two different exercise groups.

**Table 1.** Erytrocytes carbonic anhydrase spesific activity results for control, interval and continuous tarining.

Training species		Specific activity results (EU/mg protein)	% Activity
Control		0.0120 ± 0.0003	100
Interval training	1st week before training	0.0127 ± 0.0005	106
	1st week after training	0.0139 ± 0.0007	116
	2nd week after training	0.0180 ± 0.0010	150
	4th week after training	0.0128 ± 0.0004	107
	6th week after training	0.0120 ± 0.0003	100
Continuous training	1st week before training	0.0122 ± 0.0003	102
	1st week after training	0.0130 ± 0.0005	108
	2nd week after training	0.0177 ± 0.0010	148
	4th week after training	0.0135 ± 0.0006	113
	6th week after training	0.0117 ± 0.0002	98

**Table 2.** Interval running group (IRG), continuous running group (CRG) and control group (CG).

		IRG (*n* = 7)	CRG (*n* = 7)	CG (*n* = 6)
		X ± SS	X ± SS	X ± SS
Age (years)		21.6 ± 1.07	23.1 ± 2.07	24.8 ± 1.47
Length (cm)		1.77 ± 0.08	1.75 ± 0.07	1.76 ± 0.05
Body mass (kg)	Before test	69.7 ± 9.2	66.5 ± 6.0	76.8 ± 10.0
	After test	68.3 ± 9.6*	64.6 ± 5.6**	77.6 ± 9.5*
Body mass index (kg/m^2^)	Before test	21.9 ± 1.6	21.6 ± 1.6	21.9 ± 1.6
	After test	21.4 ± 1.7*	20.9 ± 1.6**	21.4 ± 1.7*
max $V_{O_{2}}$ (ml/kg/min)	Before test	42.6 ± 5.3	41.3 ± 3.0	36.7 ± 2.2
	After test	47.6 ± 4.1**	50.2 ± 3.5**	36.7 ± 1.3

*p < .05.

**p < .01.

If we consider specific activity (EU/mg prt) of the control as 100% (Table 1), no significant difference was observed in the first week between the two groups before exercise. However, 8% and 16% increase were observed in continuous group and interval group respectively after exercise. At the fourth and sixth weeks, this difference decreased to almost control value. The reason for this result may be the adaptation of the athletes in six weeks.

As for the second week, the highest values were observed both interval and continuous groups, namely, 50% and 48% increase was seen for interval and continuous groups, respectively.

A significant difference has been observed between the groups in initial and final values of body weight and body mass index (*p* < .05). Also, max $V_{O_{2}}$ values were significantly different between both test groups (*p* < .01).

## 4. Conclusions

In the present study, we investigated the effects of cold weather, high sea level and two different training methods on erythrocytes CA enzyme activities in university students. Our findings showed that CA activity initially increased fast then returned at the basal values after 4–6 weeks after the training started, probably proving a metabolic compensation without the need of an enhanced enzyme activity.
